# Effective Antiplasmodial and Cytotoxic Activities of Synthesized Zinc Oxide Nanoparticles Using *Rhazya stricta* Leaf Extract

**DOI:** 10.1155/2021/5586740

**Published:** 2021-08-26

**Authors:** Sanodia Najoom, Fozia Fozia, Ijaz Ahmad, Abdul Wahab, Nisar Ahmad, Riaz Ullah, Anadil Gul, Ahmed Bari, Muhammad Yahya Khan, Anis Ahmed Khan

**Affiliations:** ^1^Department of Chemistry, Kohat University of Science & Technology, Kohat, Pakistan; ^2^Biochemistry Department, KMU Institute of Medical Sciences, Kohat, Pakistan; ^3^Department of Pharmacy, Kohat University of Science & Technology, Kohat, Pakistan; ^4^Department of Botany, Kohat University of Science & Technology, Kohat, Pakistan; ^5^Department of Pharmacognosy, College of Pharmacy, King Saud University, Riyadh, Saudi Arabia; ^6^Beijing Key Laboratory for Green Catalysis and Separation, Beijing University of Technology, 100 PingLeYuan, Chaoyang District, Beijing 100124, China; ^7^Department of Pharmaceutical Chemistry, College of Pharmacy, King Saud University, Riyadh, Saudi Arabia; ^8^Lancaster Environmental Centre, Lancaster University, Lancaster, UK; ^9^IMU Clinical School Kluang, Kluang Johor 86000, Malaysia

## Abstract

In the present study, zinc oxide (ZnO) nanoparticles were prepared using ZnCl_2_.2H_2_O as a precursor, via green route using leaf extract of *Rhazya stricta* as capping and reducing agent. The prepared ZnO nanoparticles were examined using UV-visible spectrophotometer (UV-Vis), Fourier transform infrared spectrometer (FT-IR), X-ray diffraction spectrometer (XRD), and scanning electron microscope (SEM). The UV-Vis absorption spectrum at 355 nm showed an absorption peak, which indicates the formation of ZnO NPs. The FT-IR spectra analysis was performed to identify the potential biomolecule of the as-prepared ZnO NPs. The FT-IR spectra showed peaks at 3455, 1438, 883, and 671 cm^−1^ in the region of 4000–500 cm^−1^, which indicates –OH, NH, C-H, and M-O groups, respectively. The SEM images showed aggregation of ZnO nanoparticles with an average size of 70–90 nm. The XRD study indicated that the ZnO NPs were crystalline in nature with hexagonal wurtzite structure and broad peaks were observed at 2 theta positions 31.8°, 34.44°, 36.29°, 47.57°, 56.61°, 67.96°, and 69.07°. The synthesized ZnO NPs were found to be good antiplasmodial with a 50% inhibitory concentration (IC_50_) value of 3.41 *μ*g/mL. It is concluded from the current study that the ZnO NPs exhibited noble antiplasmodial activity, and for the improvement of antiplasmodial medications, it might be used after further *in vivo* studies.

## 1. Introduction

Nowadays, with speedy evolution of nanotechnology, many nanoparticles have been reported for applications in medical science. The nanotechnology applications in the diagnostics, healthcare sector, drug delivery, bioimaging, antimicrobial agent, and therapeutics, also referred to as nanomedicine, have enlarged ground over the past 5 years [1]. This can be perceived from the growth in the USA budget for the research of nanomedicine, as well as a rise in the number of nano-pharmaceutical patents [2].

Zinc oxide nanoparticles are one of the five zinc compounds that are presently registered and commonly believed as safe by the US Drug and Food Management. These zinc oxide nanoparticles have an antibacterial effect on Gram-positive and Gram-negative bacteria such as *Escherichia coli*, *Pseudomonas aeruginosa*, and *Staphylococcus aureus* [3, 4].

Biosynthetic and ecofriendly process for the ZnO NPs synthesis is considered being harmless, biocompatible, bio-safe in preparation of cosmetics, drug carriers, and fillings in medical materials. In comparison with silver nanoparticles, the ZnO NPs have several benefits over silver nanoparticles, such as low cost and white appearance [5, 6]. Physical and chemical methodologies are affluent for the synthesis of nanoparticles but have some limitations such as use of toxics chemicals, being costly, needing extensive effort, and being time-consuming [7]. Tremendous amounts of secondary waste are also formed from the accumulation of chemicals for reduction and precipitation in these methods. The green synthesis method using plant extract has drawn consideration as a feasible and simple alternative to physical and chemical approaches, because of its ecofriendly nature, low cost, easy handling, and wide availability of plants [8]. Zinc oxide nanoparticles have been synthesized using green seaweed *Calotropis procera, Cassia fistula*, *Melia azedarach*, and *Kalanchoe blossfeldiana* [9–11]. *Rhazya stricta* is an important medicinal plant, widely used for treatment of different diseases in Pakistan [12]. The leaf extract of *Rhazya stricta* has been used as anticancer, antifungal, analgesic, chemopreventive, rheumatism, and sedative agent [13]. Furthermore, it has many beneficial phytochemicals such as tannins, saponins, amino acids, gallic acid, and various flavonoids such as quercetin, hesperidin, kaempferol, quercetrin-3-rhamnaside, isoquercitrin, rutin, apigenin, and luteolin [14–16], which might act as reducing and stabilizing agents in synthesis of nanoparticles (NPs).

Therefore, in the current study, leaf extracts of *Rhazya stricta* were used for green synthesis of ZnO NPs, which are not yet reported.

Malaria is the most severe tropical disease of protozoan. Therefore, to eradicate malaria disease over a period, many struggles have been accomplished. But the elimination of malaria remains a faraway target, as worldwide millions of clinical cases were reported with malaria every year and over three billion people are living in its threat [17]. Malaria is caused by the plasmodium parasite, generally *P. vivax*, *P. malariae*, *P. ovale*, and *P. falciparum*. The supreme pathogenic and powerful malarial parasite among the above four species of plasmodium is *P. falciparum* in man, which spreads disease and is the vital cause of almost every malaria mortality and morbidity in tropical and subtropical countries [18]. The renowned beginning and progress of cerebral malaria are acquiescence and assumption of infected red blood cells, immune cells, and platelets to the vascular endothelial cells covering and small blood vessels of brain. In the brain, these signs lead to microhemorrhage and edema [19]. Artemisinin combination treatment for falciparum malaria are the important antimalarial medicines docile to prevalent usage against all chloroquine-resistant malaria parasites. However, malaria parasites of artemisinin-resistant were freshly perceived in Cambodia. The most recent antiplasmodial drugs from biotic reasons support in engrave this matter, but it seems to be the loss of cost-effective and biodiversity. The nanoparticles possessing various biological potential are detected as a key source of new antiplasmodial medicines and these feasible techniques were scarcely revealed [20].

The present study explores the antiplasmodial activity of ZnO NPs against plasmodium parasites in *in vitro* conditions.

## 2. Experimental

### 2.1. Extract Preparation

Fresh and healthy *Rhazya stricta* leaves were collected from the Kohat University of Science & Technology Kohat, Pakistan. The collected leaves were washed three times with distilled water and were grinded into small pieces with the help of an electrical grinder. About 10 g of grinded leaves was weighed and mixed in 100 mL of distilled water in 250 mL conical flask, followed by heating on hotplate for 15 to 20 minutes. The resultant extract was filtered through Whatman filter paper. The obtained filtrate was placed in inert plastic vials for the synthesis of nanoparticles (NPs) stored at 4°C in refrigerator.

### 2.2. Green Synthesis of ZnO NPs

For the synthesis of ZnO NPs, 1 mM solution of hydrated zinc chloride was prepared and 5 ml of leaf extract of *Rhazya stricta* to 95 ml of zinc chloride aqueous solution was added in a 250 mL conical flask. The mixture was kept on hotplate using magnetic stirrer under continuous stirring for homogeneous mixing. The flask comprising the solution was heated for 7–10 min at 80°C on water bath. The pH was maintained on 12 by adding freshly prepared NaOH (0.1 M) aqueous solution drop-wise and kept on magnetic stirrer for 1 hr to get pale white precipitates. The resultant precipitates were washed several times through distilled water, methanol, and acetone to remove any nonreacted precursors. The obtained ZnO nanoparticles precipitate was dried at 60°C in an oven, overnight [21].

### 2.3. Characterization of ZnO NPs

For the confirmation of size, shape and structure of the prepared ZnO NPs were examined by various techniques like UV-Vis, FT-IR, XRD, and SEM.

#### 2.3.1. UV-Vis Spectroscopy

A UV-Vis spectrum in the range of 300 to 600 nm of the nanoparticles was taken to initially confirm the formation of ZnO NPs using Shimadzo 1800 UV-Vis spectrophotometer.

#### 2.3.2. FT-IR Spectroscopy

FT-IR spectra were recorded in the range of 4000–500 cm^−1^ with a resolution of 4 cm^−1^, to confirm the formation of ZnO nanoparticle peaks and to find the biomolecules of the extract that are capped on ZnO NPs. FT-IR spectroscopy measurements were carried out by FT-IR spectrophotometer (Bruker-Tenson 37).

#### 2.3.3. Scanning Electron Microscopy (SEM)

The size and shape of nanoparticles were studied using SEM JSM5910 (JEOL, Japan).

#### 2.3.4. X-Ray Diffraction (XRD) Method

From XRD pattern, the average crystallite size of prepared nanoparticles was examined and also crystallinity of the particles was checked using JEOL, Japan, JDX-3532 CuK*α* (wavelength = 1.5418 Å).

#### 2.3.5. Collection of Parasite Sample

The blood of malaria-positive patient was tested in Al-Habib Clinical Laboratory Kohat, and in EDTA tubes, the blood sample of malaria positive was collected and stored at 4°C.

#### 2.3.6. Staining and Visualizing of Parasites

**Figure 1:**
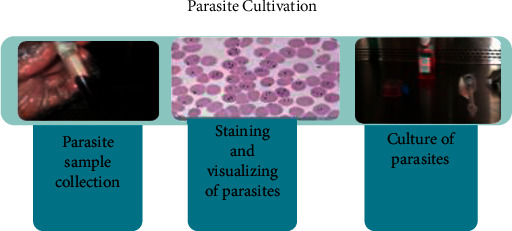
Plasmodium parasite cultivation.

Microscopic examination was accomplished to diagnose malaria on a glass slide to visualize the malaria parasite using staining thick and thin blood films shown in Figure 1. Leishman stain (0.15%) was used for staining of parasites.

#### 2.3.7. Parasites Culture

Human O Rh^+^ red blood cells of plasmodium parasites in complete RPMI 1640 medium were cultured. To the culture of plasmodium parasites, 10% inactivated serum of O Rh^+^ was added, and gentamicin sulfate, about 40 ụg mL, was also added. At 37°C parasites, culture was incubated under gas mixture of 5% O_2_, 5% CO_2_, and 90% N_2_. Every day, infected erythrocytes were transmitted into a fresh complete medium to propagate the culture [22].

#### 2.3.8. Drugs Dilution

Stock solutions of chloroquine (CQ) were prepared in Milli Q water. CQ and test compounds (plant extracts, ZnCl_2_, and ZnO NPs) were dissolved in dimethyl sulfoxide (DMSO). All stocks were then diluted with culture medium to achieve the required concentrations (in all cases except CQ, the final solution contained 0.4% DMSO, which was found to be nontoxic to the parasite). Drugs and test compounds were then placed in 96-well flat-bottom tissue-culture grade plates.

#### 2.3.9. *In Vitro* Antiplasmodial Activity

Antiplasmodial potential of the studied plant extract *Rhazya stricta*, precursor ZnCl_2_, and prepared ZnO NPs against plasmodium parasites was assessed in 96-well plates for 48 h, following the description by Okaiyeto et al. [23] using parasite lactate dehydrogenase (pLDH) activity as a biomarker. Chloroquine, an antimalarial drug, was used as a positive control. Percentage parasite viability was calculated relative to the pLDH activity in wells containing untreated control parasites.

### 2.4. Cytotoxicity Examination by Hemolysis

Cytotoxicity analysis of synthesized nanoparticles is an essential step to check its biocompatibility. Thus, we carried out the hemolysis test of synthesized ZnO NPs by using reported method [24, 25]. Briefly, blood (9 mL) was collected from goat for the experiment and 1 mL (3.8%) sodium citrate added to it to prevent coagulation. Then, the obtained blood was centrifuged for 8 min at 3000 rpm, the supernatant was removed, and the obtained pellet (RBC) was dispersed in 10 mL PBS (phosphate buffer saline), and the same process was repeated 3 times to remove the buffy coat of RBC completely. After complete removal of buffy coat, the obtained RBC were dispersed in PBS; then 2 mL of the suspension was added in 5 test tubes each. Afterwards, different doses (0.25, 0.5, 1.0, 2.5, and 5.0 mg/mL) of ZnO nanoparticles were added in the above 5 test tubes and the tubes were shaken to mix the cells and NPs and then incubated at 30°C for 90 min. After 90 min, the mixture was centrifuged for 8 min at 3000 rpm and the supernatant was examined by UV-Vis spectrophotometer (*λ* = 540 nm). 2 mL of RBC suspension was mixed with PBS (pH 7.4) and Triton X-100 for negative and positive control.

The hemolysis percentage was calculated by using the following formula:

(1) $$\begin{array}{c} \%\text{ hemolysis}=\frac{{\text{OD}}_{\text{test sample }}-{\text{OD}}_{\text{negative control}}}{{\text{OD}}_{\text{postive control}}\;-\;{\text{OD}}_{\text{negative control}}}\times 100. \end{array}$$

The optical density of negative was 0.005 and positive control was 1.302.

## 3. Results and Discussion

### 3.1. UV-Vis Spectroscopy Study of ZnO NPs

**Figure 2:**
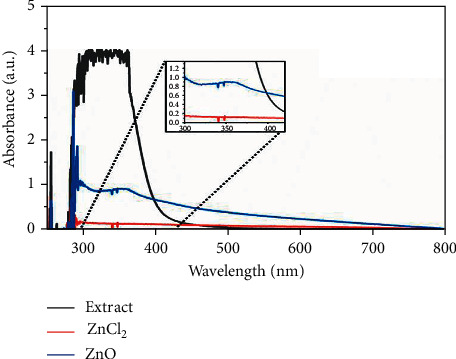
UV-Vis spectra of the synthesized ZnO NPs/ZnCl_2_ and plant extract.

A UV-Vis spectrum was taken in the range of 300–600 nm of the prepared ZnO NPs. Peak at 353 nm indicates the formation of ZnO NPs in Figure 2 [26]. The UV-Vis spectral analysis of leaf extract of *Rhazya stricta* and the precursor ZnCl_2_ is also shown in Figure 2.

### 3.2. FT-IR Studies of the ZnO NPs

**Figure 3:**
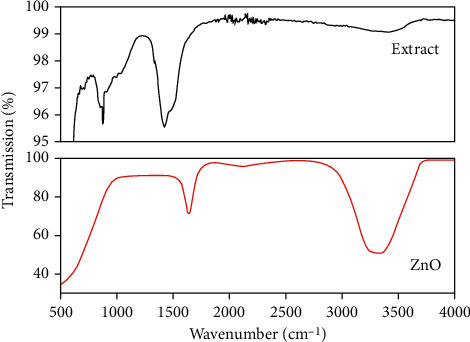
FT-IR spectra of plant extract and the synthesized ZnO NPs.

The FT-IR spectra of *Rhazya stricta* aqueous extract as shown in Figure 3 showed three main absorption bands at 3362, 2164, and 1654 cm^−1^. The absorption band at 3362 cm^−1^ could be attributed to the O‒H stretching vibration of flavonoids/saponins, and the band at 2164 cm^−1^ shows C-N stretching; the band at 1654 cm^−1^ shows C=O stretching vibrations of amine.

The functional groups in leaf extract of *Rhazya stricta* identified by FT-IR were used as a stabilizing and capping agent in the preparation of ZnO nanoparticles.

The FT-IR studies of biogenic ZnO NPs also showed a similar arrangement of absorption bands with certain degrees shift in the band's positions, specifying a possible action of phenolic compounds and flavonoids which capped ZnO NPs. Figure 3 also shows the band near the 672 cm^−1^ indicating metal oxide stretching vibration for ZnO nanoparticles. However, the bands at 3472 cm^−1^ and 1435 cm^−1^ are the characteristics peaks of hydroxyl [27] and amine [28] groups, respectively; the band at 882 cm^−1^ may be assigned to carbon-hydrogen bending vibrations [29].

### 3.3. SEM Studies of ZnO NPs

**Figure 4:**
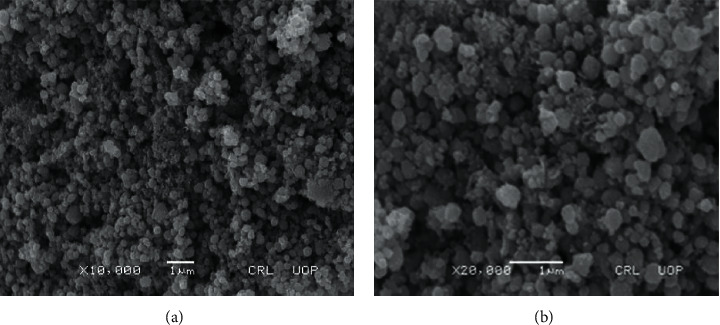
SEM images (a, b) of ZnO NPs under different magnifications.

The formations of ZnO NPs, as well as their morphological extents, were studied by using SEM technique. The surface morphology of the ZnO nanoparticles under different magnifications is shown in Figures 4(a) and 4(b). The current study confirmed that the average size of the particle was found to be 70–90 nm with random spherical shape. Moreover, particles were aggregated and poly-dispersed.

### 3.4. XRD Studies

**Figure 5:**
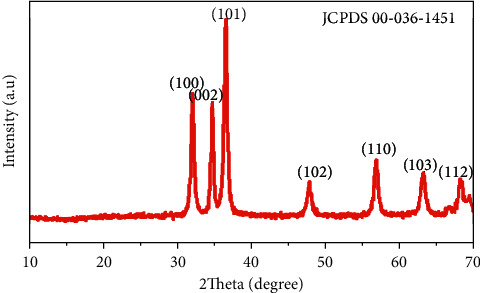
XRD patterns of the newly synthesized ZnO NPs.

Figure 5 shows the XRD pattern of the biosynthesized ZnO NPs using *Rhazya stricta* leaf extract. All X-ray diffraction peaks found at 31.8°, 34.44°, 36.29°, 47.57°, 56.61°, 67.96°, and 69.07° present that the synthesized ZnO nanoparticles are in analogous to the representative Bragg peaks (1 0 0), (0 0 2), (1 0 1), (1 0 2), (1 1 0), (1 1 2), (2 0 1) planes of a ZnO hexagonal wurtzite structure [30] and are in the good arrangement of the synthesized ZnO nanoparticles with standard diffraction data available in the library (JCPDS 00-036-1451). This undoubtedly confirms that new synthesized ZnO NPs have been effectively prepared by the green synthesis route.

The peaks of XRD with high intensities suggested that the synthesized ZnO NPs were highly crystalline using leaf extract of *Rhazya stricta*.

The crystalline size of the biosynthesized ZnO NPs using the peak at 36. 405 (101 was reported 18.96 by Scherer's formula) [26].

### 3.5. Antiplasmodial Activity

The results of the antiplasmodial activity leaf extract of *Rhazya stricta*, precursor ZnCl_2_, and ZnO NPs are depicted in Table 1. A dose-response assay yielded an antiplasmodial IC_50_ value of 19.3 *μ*g/mL, 11.6 *μ*g/mL, and 3.41 *μ*g/mL for the leaf extract of *Rhazya stricta*, ZnCl_2_, and ZnO NPs, respectively.

It is clear from the inhibition concentration (IC_50_) values that plant extracts show low antiplasmodial activity, ZnCl_2_ shows moderate antiplasmodial activity, and ZnO NPs shows highest antiplasmodial activity against plasmodium parasites. So it was concluded that ZnO NPs are good antiplasmodial agents.

## 4. Discussion

ZnO NPs synthesis by physical methods is either expensive, lengthy, or time-wasting, whereas chemical methods frequently cause poisonous waste making them unfavorable environment. Furthermore, occasionally harmful chemicals on the surface of the nanoparticles can be adsorbed, which cannot be useful in medical purposes [31]. The progress of stimulated biologically experimental routes for the nanoparticle synthesis is grown into an essential nanotechnology branch. The current research work highlights the medicinal plant use for the ZnO NPs biosynthesis with effective antiplasmodial activity against plasmodium parasites.

ZnO NPs were obtained using *Rhazya Stricta* leaf extract, which were collected from strong and vigorous plants, comprising the stabilizing and capping agents. During the reaction, the mixture color was changed to pale white from green-yellow, indicating the ZnO NPs formation. It was observed that the leaf extract of *Rhazya stricta* containing electron-rich functional groups accounted for the formation of ZnO NPs. Therefore, it was determined that the extract of *Rhazya stricta* leaf for the ZnO NPs formation acts as a capping and stabilizing agent.

Different techniques for the confirmation of the prepared ZnO nanoparticles were used.

The most commonly used technique for the nanoparticles structural characterization is UV-Vis spectroscopy. In Figure 2, UV-Vis spectra show the absorbance peak at 353 analogous to the specific band of ZnO NPs [26].

The existence of hydroxyl and amine group in FT-IR spectra of *Rhazya stricta* indicates that it is used as a stabilizing and capping agent in the synthesis of ZnO NPs.

The SEM studies under different magnification (Figures 4(a) and 4(b)) of the synthesized ZnO NPs exhibit relatively random spherical shape of nanoparticle and size in the range of 70–90 nm. Similarly, they illustrate distinguished random sphere-shaped particles, which is analogous to those explored in the previously reported literature [26].

The XRD pattern of the synthesized nanoparticles confirms that ZnO nanoparticles are hexagonal wurtzite crystal structure. The crystalline size of the biosynthesized ZnO NPs was found to be 18.96 nm calculated by Scherrer's formula. The results of the current study also contest with the work reported by S. P. R. Verma et al. [26].

Parasitic diseases (like malaria, leishmaniasis, and trypanosomiasis) around the earth are one of the chief problems [32, 33]. Malaria is a life-threatening disease caused by parasites that are transmitted to people through the bites of infected female *Anopheles* mosquitoes [34]. In 2017, malaria cases from 87 countries were estimated to be about 219 million [35]. Traditional medicines temporarily have been used for thousands of years to treat malaria. The plant is usually used in traditional treatment in Kenya to control malaria [36]. Nanoparticles have presented an innovative approach for drug delivery efficiency because of their reduced size and large surface-area-to-volume ratio, which aids their ability to penetrate the cell membrane [37]. The development of effective and reliable drugs in the fight against malaria represents a crucial challenge in modern parasitology [38]. Phytochemical compounds that demonstrate high antiplasmodial activity could effectively serve as an alternative to synthetic antimalarial drugs [39].

In order to know the medicinal potential of ZnO NPs prepared by using leaf extract of *Rhazya stricta*, we determined their antiplasmodial activity to inhibit the plasmodium parasites *in vitro* condition. These ZnO NPs were found to be good antiplasmodial with the highest IC_50_ value 3.41 *μ*g/mL. The leaf extract of *Rhazya stricta* also showed low antiplasmodial activity with IC_50_ value 19.3 *μ*g/mL followed by ZnCl_2_, which showed moderate antiplasmodial activity with IC_50_ value 11.6 *μ*g/mL.

The present study is compared with some already reported literature and is summarized in Table 2.

It was confirmed from the current study that ZnO NPs stabilized with leaf extract of *Rhazya Stricta* change the plasmodium parasite's action. The results openly indicate that ZnO NPs are good plasmodium growth inhibitors illustrating good antiplasmodial activity.

ZnO NPs are extensively used in the biomedical field due to their antibacterial, antifungal, and antiplasmodial activity; hence, the biocompatibility knowledge of ZnO NPs is essential. For the transfer of any type of drugs or NPs to targeted body organ, the blood is the primary carrier [45]. Thus, the biocompatibility of ZnO NPs with blood is more important for its practical application.  Table 3 demonstrates the hemolysis % of ZnO NPs at different concentrations (0.25, 0.5, 1.0, 2.5, and 5.0 mg); the hemolysis % shows increasing phenomenon with increasing the dose of ZnO NPs.

It can be seen from Table 3 that ZnO NPs show 4.43% hemolysis by using 2.5 mg dose (ZnO NPs), which is within the permissible limit as the permissible limit for biomaterials according to ASTM-E252408 is 5% hemolysis [46]. However, at 5 mg dose of ZnO NPs, the % hemolysis is 9.68%, which is above the permissible limit; thus we can conclude that the synthesized ZnO NPs show good biocompatibility up to 2.5 mg dose (ZnO NPs). The obtained results are in good agreement with reported literature [25].

**Figure 6:**
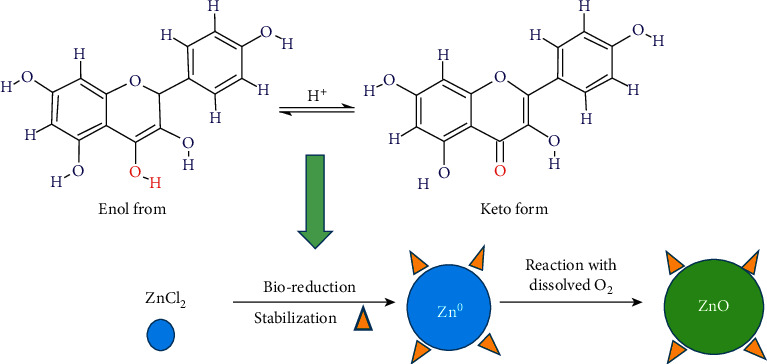
Proposed mechanism for the synthesis of ZnO NPs.

According to reported literature, the leaf extract of *Rhazya stricta* contained various compounds like tannins, saponins, amino acids, gallic acid, and various flavonoids such as quercitrin, hesperidin, kaempferol, quercetrin-3-rhamnaside, isoquercitrin, rutin, apigenin, and luteolin [14–16]. The exact mechanism of metal (ZnO) NPs using plants extract is unobvious due to the complex chemicals composition of plant extract; however, the possible mechanism is proposed in view of the above chemicals composition of *Rhazya stricta.* Hence, Figure 6 shows the possible mechanism for synthesis of ZnO NPs using leaves extract of *Rhazya stricta.*

The flavonoid is converted from enol form into keto form and produces reactive hydrogen. The produce reactive hydrogen acts as a reducing agent, converting Zn^+2^ into Zn^0^, which further reacts with O_2_ (dissolved) and forms ZnO NPs. Further, the amine and flavonoid act as capping agent and stabilize the synthesized ZnO NPs. Mohammadi et al. [47] and Matinise et al. [48] also proposed such type of mechanism for the synthesis of ZnO NPs. Thus, the synthesis of metal NPs by using plants extract has much more benefits as compared to chemical route. The plant extract plays dual role as reducing as well as stabilizing agent, reducing the use of harm chemicals, being environmentally friendly, easily available, and cost-effective as compared to chemical route [49].

## 5. Conclusion

The fast-biological synthesis of zinc oxide nanoparticles using leaf extract of *Rhazya stricta* provides an environmentally friendly, simple, and efficient route for synthesis of nanoparticles.

In the current study, ZnO NPs with crystallite size of 18.96 nm were effectively synthesized using leaf extract of *Rhazya stricta*. The confirmation of the prepared ZnO NPs had been characterized by UV-Vis spectroscopy, FT-IR spectroscopy, SEM, and XRD techniques. The results showed that the prepared ZnO NPs inhibited significantly the growth of plasmodium parasites with the highest IC_50_ value 3.41 *μ*g/mL, which specified ZnO NPs are good antiplasmodial agents against plasmodium parasites. Furthermore, the synthesized NPs showed good biocompatibility, as they showed only 4.43% hemolysis with maximum dose.

## Figures and Tables

**Table 1:** In vitro antiplasmodial activity of plant extract/ZnCl_2_ and the synthesized ZnO NPs.

S. no	Test samples	Concentration of test samples (*μ*g/mL)	IC_50_ value (*μ*g/mL)
1	Plant extract *Rhazya stricta*	50	19.3
2	ZnCl_2_	50	11.6
3	ZnO NPs	50	3.41
4	Chloroquine (positive control)	50	0.026

**Table 2:** Comparison of the present study with published literature.

S. no	Nanoparticles/plant extract	IC_50_ value (*μ*g/mL)	Reference
1	AgNPs/*Azadirachta indica* and *Ocimum sanctum*	35–40	[40]
2	AgNPs/*Callistemon citrinus*	2.99–5.34	[41]
3	TiO_2_ NPs/*Momordica charantia*	53.42 (CQ-s) and 59.71 (CQ-r)	[42]
4	EW-AgNP (earth worm-mediated silver nanoparticles)	49.3 (CQ-s) and 55.5 (CQ-r)	[43]
5	Ag NPs/amylase Ag NPs/Ashoka plant extract Ag NPs/neem plant extract	3.75 8 30	[44]
6	ZnO ZnCl_2_ Leaf extract of *Rhazya stricta*	3.41 11.6 19.3	This study

**Table 3:** Hemolysis test by using synthesized ZnO NPs.

S. no	Sample (mg)	OD^*∗*^ at 540 nm	Hemolysis (%)
1	Triton X-100	1.297 ± 0.0002	+ve control
2	PBS	1.48 ± 0.0001	−ve control
3	0.25	0.0125 ± 0.0004	0.59
4	0.5	0.025 ± 0.0002	1.56
5	1.0	0.044 ± 0.001	3.0
6	2.5	0.062 ± 0.003	4.43
7	5.0	0.13 ± 0.002	9.68

## Data Availability

The data used to support the findings of this study are included within the article.

## References

[1] Nasrollahzadeh M., Sajjadi M., Dadashi J., Ghafuri H. (2020). Pd-based nanoparticles: plant-assisted biosynthesis, characterization, mechanism, stability, catalytic and antimicrobial activities. *Advances in Colloid and Interface Science*.

[2] Mishra B., Patel B. B., Tiwari S. (2010). Colloidal nanocarriers: a review on formulation technology, types and applications toward targeted drug delivery. *Nanomedicine: Nanotechnology, Biology and Medicine*.

[3] Emami-Karvani Z., Chehrazi P. (2011). Antibacterial activity of ZnO nanoparticle on gram-positive and gram-negative bacteria. *African Journal of Microbiology Research*.

[4] Gul A., Fozia A., Shaheen A. (2021). Green synthesis, characterization, enzyme inhibition, antimicrobial potential, and cytotoxic activity of plant mediated silver nanoparticle using Ricinus communis leaf and root extracts. *Biomolecules*.

[5] Rosi N. L., Mirkin C. A. (2005). Nanostructures in biodiagnostics. *Chemical Reviews*.

[6] Bordbar M., Negahdar N., Nasrollahzadeh M. (2018). *Melissa Officinalis* L. leaf extract assisted green synthesis of CuO/ZnO nanocomposite for the reduction of 4-nitrophenol and Rhodamine B. *Separation and Purification Technology*.

[7] Hatamifard A., Nasrollahzadeh M., Lipkowski J. (2015). Green synthesis of a natrolite zeolite/palladium nanocomposite and its application as a reusable catalyst for the reduction of organic dyes in a very short time. *RSC Advances*.

[8] Faisal S., Jan H., Shah S. A. (2021). Green synthesis of zinc oxide (ZnO) nanoparticles using aqueous fruit extracts of *Myristica fragrans*: their characterizations and biological and environmental applications. *ACS Omega*.

[9] Singh R. P., Shukla V. K., Yadav R. S., Sharma P. K., Singh P. K., Pandey A. C. (2011). Biological approach of zinc oxide nanoparticles formation and its characterization. *Advanced Materials Letters*.

[10] Naseer M., Aslam U., Khalid B., Chen B. (2020). Green route to synthesize Zinc Oxide Nanoparticles using leaf extracts of *Cassia fistula* and *Melia azadarach* and their antibacterial potential. *Scientific Reports*.

[11] Aldalbahi A., Alterary S., Ali Abdullrahman Almoghim R. (2020). Greener synthesis of zinc oxide nanoparticles: characterization and multifaceted applications. *Molecules*.

[12] Bukhari N. A., Al-Otaibi R. A., Ibhrahim M. M. (2017). Phytochemical and taxonomic evaluation of Rhazya stricta in Saudi Arabia. *Saudi Journal of Biological Sciences*.

[13] Khan S. A., Baeshen M. N., Ramadan H. A., Baeshen N. A. (2017). Emergence of plastidial intergenic spacers as suitable DNA barcodes for arid medicinal plant rhazya stricta. *American Journal of Plant Sciences*.

[14] Baeshen M. N., Khan R., Bora R. S., Baeshen N. A. (2015). Therapeutic potential of the folkloric medicinal plant Rhazya stricta. *Biological Systems: Open Access*.

[15] Lanjwani A. H., Ganghro A. B., Khuhawar T. M. J. (2018). Phytochemical analysis and biological activity of diffirent parts of rhazya stricta. *Rawal Medical Journal*.

[16] Al-Dabbagh B., Elhaty I. A., Al Sakkaf R., El-Awady R., Ashraf S. S., Amin A. (2018). Antioxidant and anticancer activities of Trigonella foenum-graecum, Cassia acutifolia and Rhazya stricta. *BMC Complementary and Alternative Medicine*.

[17] Snow R. W., Guerra C. A., Noor A. M., Myint H. Y., Hay S. I. (2005). The global distribution of clinical episodes of *Plasmodium falciparum* malaria. *Nature*.

[18] Tuteja R. (2007). Malaria—an overview. *FEBS Journal*.

[19] Hunt N. H., Grau G. E. (2003). Cytokines: accelerators and brakes in the pathogenesis of cerebral malaria. *Trends in Immunology*.

[20] Jacob Inbaneson S., Ravikumar S. (2013). In vitro antiplasmodial activity of PDDS-coated metal oxide nanoparticles against *Plasmodium falciparum*. *Applied Nanoscience*.

[21] Nagarajan S., Arumugam Kuppusamy K. (2013). Extracellular synthesis of zinc oxide nanoparticle using seaweeds of gulf of Mannar, India. *Journal of Nanobiotechnology*.

[22] Panneerselvam C., Ponarulselvam S., Murugan K. (2011). Potential anti-plasmodial activity of synthesized silver nanoparticle using *Andrographis paniculata* Nees (Acanthaceae). *Archives of Applied Science Research*.

[23] Okaiyeto K., Hoppe H., Okoh A. I. (2021). Plant-based synthesis of silver nanoparticles using aqueous leaf extract of *Salvia officinalis*: characterization and its antiplasmodial activity. *Journal of Cluster Science*.

[24] Miki M., Tamai H., Mino M., Yamamoto Y., Niki E. (1987). Free-radical chain oxidation of rat red blood cells by molecular oxygen and its inhibition by *α*-tocopherol. *Archives of Biochemistry and Biophysics*.

[25] Das D., Nath B. C., Phukon P., kalita A., Dolui S. K. (2013). Synthesis of ZnO nanoparticles and evaluation of antioxidant and cytotoxic activity. *Colloids and Surfaces B: Biointerfaces*.

[26] Verma P. R., Khan F., Banerjee S. (2020). Salvadora persica root extract-mediated fabrication of ZnO nanoparticles and characterization. *Inorganic and Nano-Metal Chemistry*.

[27] Khodadadi B., Bordbar M., Nasrollahzadeh M. (2017). *Achillea millefolium* L. extract mediated green synthesis of waste peach kernel shell supported silver nanoparticles: application of the nanoparticles for catalytic reduction of a variety of dyes in water. *Journal of Colloid and Interface Science*.

[28] Nasrollahzadeh M., Mohammad Sajadi S., Rostami-Vartooni A. (2015). Green synthesis of CuO nanoparticles by aqueous extract of Anthemis nobilis flowers and their catalytic activity for the A3 coupling reaction. *Journal of Colloid and Interface Science*.

[29] Dobrucka R., Długaszewska J. (2016). Biosynthesis and antibacterial activity of ZnO nanoparticles using *Trifolium pratense* flower extract. *Saudi Journal of Biological Sciences*.

[30] Ahmad M., Rehman W., Khan M. M. (2021). Phytogenic fabrication of ZnO and gold decorated ZnO nanoparticles for photocatalytic degradation of Rhodamine B. *Journal of Environmental Chemical Engineering*.

[31] Nain V., Kaur M., Sandhu K. S., Thory R., Sinhmar A. (2020). Development, characterization, and biocompatibility of zinc oxide coupled starch nanocomposites from different botanical sources. *International Journal of Biological Macromolecules*.

[32] Edwards G., Krishna S. (2004). Pharmacokinetic and pharmacodynamic issues in the treatment of parasitic infections. *European Journal of Clinical Microbiology & Infectious Diseases*.

[33] Reidpath D. D., Allotey P., Pokhrel S. (2011). Social sciences research in neglected tropical diseases 2: a bibliographic analysis. *Health Research Policy and Systems*.

[34] Udayabhanu J., Kannan V., Tiwari M., Natesan G., Giovanni B., Perumal V. (2018). Nanotitania crystals induced efficient photocatalytic color degradation, antimicrobial and larvicidal activity. *Journal of Photochemistry and Photobiology B: Biology*.

[35] World Health Organization (2019). World malaria report. https://www.who.int/news-room/fact-sheets/detail/malaria.

[36] Muregi F. W., Chhabra S. C., Njagi E. N. M. (2003). In vitro antiplasmodial activity of some plants used in Kisii, Kenya against malaria and their chloroquine potentiation effects. *Journal of Ethnopharmacology*.

[37] Kumar D., Kumar G., Das R., Agrawal V. (2018). Strong larvicidal potential of silver nanoparticles (AgNPs) synthesized using Holarrhena antidysenterica (L.) Wall. bark extract against malarial vector, *Anopheles stephensi* Liston. *Process Safety and Environmental Protection*.

[38] Kamaraj C., Balasubramani G., Siva C. (2017). Ag nanoparticles synthesized using *β*-caryophyllene isolated from murraya koenigii: antimalarial (plasmodium falciparum 3D7) and anticancer activity (A549 and HeLa cell lines). *Journal of Cluster Science*.

[39] Govindarajan M., Rajeswary M., Veerakumar K., Muthukumaran U., Hoti S. L., Benelli G. (2016). Green synthesis and characterization of silver nanoparticles fabricated using Anisomeles indica: mosquitocidal potential against malaria, dengue and Japanese encephalitis vectors. *Experimental Parasitology*.

[40] Sardana M., Agarwal V., Pant A., Kapoor V., Pandey K. C., Kumar S. (2018). Antiplasmodial activity of silver nanoparticles: a novel green synthesis approach. *Asian Pacific Journal of Tropical Biomedicine*.

[41] Larayetan R., Ojemaye M. O., Okoh O. O., Okoh A. I. (2019). Silver nanoparticles mediated by Callistemon citrinus extracts and their antimalaria, antitrypanosoma and antibacterial efficacy. *Journal of Molecular Liquids*.

[42] Rajiv Gandhi P., Jayaseelan C., Kamaraj C., Rajasree S. R., Mary R. R. (2018). In vitro antimalarial activity of synthesized TiO_2_ nanoparticles using Momordica charantia leaf extract against Plasmodium falciparum. *Journal of Applied Biomedicine*.

[43] Jaganathan A., Murugan K., Panneerselvam C. (2016). Earthworm-mediated synthesis of silver nanoparticles: a potent tool against *hepatocellular carcinoma, Plasmodium falciparum* parasites and malaria mosquitoes. *Parasitology International*.

[44] Mishra A., Kaushik N. K., Sardar M., Sahal D. (2013). Evaluation of antiplasmodial activity of green synthesized silver nanoparticles. *Colloids and Surfaces B: Biointerfaces*.

[45] Li S.-Q., Zhu R.-R., Zhu H. (2008). Nanotoxicity of TiO2 nanoparticles to erythrocyte in vitro. *Food and Chemical Toxicology*.

[46] Chen L. Q., Fang L., Ling J., Ding C. Z., Kang B., Huang C. Z. (2015). Nanotoxicity of silver nanoparticles to red blood cells: size dependent adsorption, uptake, and hemolytic activity. *Chemical Research in Toxicology*.

[47] Mohammadi C., Mahmud S., Abdullah S. M., Mirzae Y. (2017). Green synthesis of ZnO nanoparticles using the aqueous extract of Euphorbia petiolata and study of its stability and antibacterial properties. *Moroccan Journal of Chemistry*.

[48] Matinise N., Fuku X. G., Kaviyarasu K., Mayedwa N., Maaza M. (2017). ZnO nanoparticles via *Moringa oleifera* green synthesis: physical properties & mechanism of formation. *Applied Surface Science*.

[49] Singh J., Dutta T., Kim K. H., Rawat M., Samddar P., Kumar P. (2018). Green synthesis of metals and their oxide nanoparticles: applications for environmental remediation. *Journal of Nanobiotechnology*.

